# Lower Functional Connectivity of the Periaqueductal Gray Is Related to Negative Affect and Clinical Manifestations of Fibromyalgia

**DOI:** 10.3389/fnana.2017.00047

**Published:** 2017-06-08

**Authors:** Marie-Andrée Coulombe, Keith St. Lawrence, Dwight E. Moulin, Patricia Morley-Forster, Mahsa Shokouhi, Warren R. Nielson, Karen D. Davis

**Affiliations:** ^1^Division of Brain, Imaging and Behaviour-Systems Neuroscience, Krembil Research Institute, Toronto Western Hospital, University Health NetworkToronto, ON, Canada; ^2^Lawson Health Research InstituteLondon, ON, Canada; ^3^Department of Medical Biophysics, University of Western OntarioLondon, ON, Canada; ^4^Departments of Clinical Neurosciences and Oncology, University of Western OntarioLondon, ON, Canada; ^5^Department of Anesthesia and Perioperative Medicine, University of Western OntarioLondon, ON, Canada; ^6^Department of Psychology, University of Western OntarioLondon, ON, Canada; ^7^Department of Surgery and Institute of Medical Science, University of TorontoLondon, ON, Canada

**Keywords:** functional magnetic resonance imaging, periaqueductal gray, resting-state fMRI, functional connectivity, descending pain modulation, fibromyalgia

## Abstract

Fibromyalgia (FM) syndrome is characterized by chronic widespread pain, muscle tenderness and emotional distress. Previous studies found reduced endogenous pain modulation in FM. This deficiency of pain modulation may be related to the attributes of chronic pain and other clinical symptoms experienced in patients with FM. Thus, we tested whether there is a link between the clinical symptoms of FM and functional connectivity (FC) of the periaqueductal gray (PAG), a key node of pain modulation. We acquired resting state 3T functional MRI (rsfMRI) data from 23 female patients with FM and 16 age- and sex- matched healthy controls (HC) and assessed FM symptoms with the Brief Pain Inventory (BPI), Fibromyalgia Impact Questionnaire (FIQ), Hospital Anxiety and Depression Scale (HADS) and Pain Catastrophizing Scale (PCS). We found that patients with FM exhibit statistically significant disruptions in PAG FC, particularly with brain regions implicated in negative affect, self-awareness and saliency. Specifically, we found that, compared to HCs, the FM patients had stronger PAG FC with the lingual gyrus and hippocampus but weaker PAG FC with regions associated with motor/executive functions, the salience (SN) and default mode networks (DMN). The attenuated PAG FC was also negatively correlated with FIQ scores, and positively correlated with the magnification subscale of the PCS. These alterations were correlated with emotional and behavioral symptoms of FM. Our study implicates the PAG as a site of dysfunction contributing to the clinical manifestations and pain in FM.

## Introduction

Fibromyalgia (FM) is a chronic pain condition present in 2%–8% of the population with a higher prevalence in women (Clauw, 2014). Patients with FM suffer tremendously not only from chronic widespread pain but also from muscle tenderness, persistent fatigue, sleep disturbances, mood and cognitive changes, the cause of which are not understood (Wolfe et al., 1990, 2010; Clauw, 2014).

The peripheral and central mechanisms underlying FM are not fully known. Recent studies of skin biopsies (Levine and Saperstein, 2015), found that patients with small fiber polyneuropathy (SFPN) had reduced small fiber diameters compared to healthy subjects (Doppler et al., 2015), and this in consistent with decreased C fiber nociceptors conduction velocity by Serra et al. (2014). However, SFPN was only observed in 30%–50% of patient previously diagnosed with FM and has also been found in some healthy controls (HC; Oaklander et al., 2013; Giannoccaro et al., 2014; Serra et al., 2014). Furthermore, decreased intraepidermal nerve fiber density can be found in other conditions (Devigili et al., 2008) and there is no correlations between small fiber diameters and symptoms severity (Kosmidis et al., 2014; Doppler et al., 2015). Therefore, the role of the peripheral and central nervous systems in FM is still not understood. Given that objective measures of small fibers does not necessarily correlate with perceived pain in FM, the central nervous system and its neuroplasticity is likely to play some role in FM (Clauw, 2015). In support of a central contribution to FM, several psychophysical studies point to abnormal central processing of nociceptive inputs and ineffective descending modulation of nociceptive signals. For example, patients with FM have lower pain thresholds, enhanced temporal summation (Price et al., 2002; Staud and Smitherman, 2002; Staud et al., 2003; Serra et al., 2014), and deficient conditioned pain modulation (Lautenbacher and Rollman, 1997; Julien et al., 2005). Furthermore, a functional MRI (fMRI) study reported brain regions involved in descending pain modulation such as the periaqueductal gray (PAG) have attenuated responses to painful stimuli in FM patients (Jensen et al., 2012).

The PAG is known for its role in both acute and chronic pain and analgesia (Reynolds, 1969; Dostrovsky and Deakin, 1977; Lovick, 1985; Keay and Bandler, 1993; Hemington and Coulombe, 2015), but it is also involved in fear, anxiety and cardiovascular responses (Bandler et al., 1985, 2000; Bandler and Shipley, 1994; Linnman et al., 2012). These functions are particularly important for emotional and behavioral responses to stress and pain. Previous studies suggested that the PAG is an integration center that generates an appropriate behavioral and autonomic response to stress and pain.

We recently studied functional connectivity (FC), i.e., synchronous slow frequency oscillation between brain areas (Davis and Moayedi, 2013), of the PAG. We reported that subregions of the PAG has FC not only with brain regions involved in descending pain modulation (rACC, aMCC, medulla), but also regions related to executive functions, such as the prefrontal cortex (PFC), striatum and the hippocampus (Coulombe et al., 2016). This information allows us to infer that these brain regions are working as a network and its dysfunction could lead to clinical signs and symptoms. The PAG shows abnormal FC in many chronic pain diseases, including FM (Cifre et al., 2012; Pujol et al., 2014). Considering these observations, and previous animal research, the PAG likely is involved not only in shaping pain perception, but also in coping behavior related to an unavoidable pain, extended to social interactions worrying and catastrophizing under conditions of chronic pain (Hassett et al., 2000; Cifre et al., 2012; Pujol et al., 2014; Coulombe et al., 2016).

Therefore, the aim of this study was to measure resting state FC of the PAG in FM and HC and link abnormalities with FM clinical symptoms (measured using standardized questionnaires (*Fibromyalgia Impact Questionnaire (FIQ)*, *Hospital Anxiety and Depression Scale (HADS), Pain Catastrophization Scale)*. We tested the hypothesis that there is abnormal PAG FC in patients with FM that are related to their chronic pain symptoms and affect-related clinical symptoms. Patients with FM not only suffer from chronic pain but often report depression, anxiety, catastrophization and cognition impairment which can diminish their quality of life (Hassett et al., 2000). Because hypervigilance has been suggested in this pathology (Crombez et al., 2004; Eccleston and Crombez, 2007), we expected FC with brain region such as the default mode and salience networks (DMN, SN) to be related to FM clinical symptoms. These networks are known to be disrupted in many chronic pain diseases, including in FM (Baliki et al., 2008; Napadow et al., 2010; Loggia et al., 2013; Kucyi et al., 2014).

## Materials and Methods

### Participants

Twenty-three female FM patients and 16 age- and sex- matched HC were recruited. Patients were recruited through the Rheumatology Clinic, St. Joseph’s Health Care London, Canada, and were diagnosed with FM using the criteria of the *American College of Rheumatology* (Wolfe et al., 1990). They were also screened for any exclusion criteria, i.e., a concurrent treatment with antidepressants, an active psychosis, or any recent change in their pain medication (any dose alternation within the preceding month). The HC subjects were recruited from the community. They were screened and excluded if they were pregnant or breastfeeding, suffering from chronic illness including neurological/psychiatric disorders. All subjects provided informed written consent to procedures approved by the Health Sciences Research Ethics Board of the University of Western Ontario. As previously described (Shokouhi et al., 2016). FM patients and HC did not differ in terms of age (FM: 50.6 ± 8.1 years; HC: 49.8 ± 11.0 years; Mann-Whitney U statistic = 177.5, *p* = 0.864). Arterial spin labeling (ASL) data from these study participants have been previously reported (Shokouhi et al., 2016).

We used standardized questionnaires in order to evaluate their pain and other collateral symptoms, such as anxiety, depression, catastrophization and impact of the disease on their life. Clinical assessment was performed only in FM subjects and included validated and commonly used metrics: the severity of symptoms in FM was evaluated using the self-administered *FIQ*, which is a well-known measure of functional disabilities such as pain, stiffness, fatigue, anxiety, depression, physical functioning, work status and well-being in FM (Wolfe et al., 1997; Perrot et al., 2003; Bennett, 2005). Depressive mood and anxiety disorders were assessed using the *HADS* (Zigmond and Snaith, 1983). Pain severity and impact of pain on daily functions were assessed using the self-administered* Brief Pain Inventory* (BPI; Cleeland and Ryan, 1994; Atkinson et al., 2011). The Pain catastrophizing scale (PCS) evaluates three dimensions of catastrophic thinking related to pain: rumination, magnification, and helplessness (Sullivan et al., 1995). The pain disability index (PDI) measures the impact of pain on a person’s essential life activities such as family/home responsibilities, recreation, social activity, occupation, sexual behavior, self-care and life-supporting activities (Tait et al., 1987). Each area is rated on an 11-point scale (0 = no disability to 10 = total disability) for a maximum score of 70 (34). All questionnaires have good internal consistency and construct validity and for all of them a higher score indicates a worse condition.

### Image Acquisition

Each participant underwent neuroimaging on a 3T MRI system (Biograph mMR, Siemens, Erlanger, Germany) at the Lawson Health Research Institute with an 32-element receive-only head coil, including a high-resolution T1-weighted whole-brain anatomical scan (176 axial slices; 256 × 256 matrix; 1 × 1 × 1 mm voxels) using a 3-D magnetization-prepared rapid gradient-echo imaging sequence (flip angle = 9°; TE = 2.98 ms; TR = 2000 ms; TI = 900 ms), and a 10.5-min resting state fMRI T2* weighted gradient echo echo-planar imaging sequence (GRE-EPI) scan (42 slices; 200 × 200 matrix; 2 × 2 × 3 mm voxels; TE = 30 ms; TR = 3000 ms; 210 volumes). Subjects were instructed to stay awake with their eyes closed (Shokouhi et al., 2016).

### Images Pre-Processing

All images were preprocessed using standard methods previously published, using freely available FSL software (FMRIB‘s software library^1^), Matlab customized scripts, and fMRISTAT toolbox. Our preprocessing steps included the deletion of the first four volumes, the removal of non-brain tissue (FSL’s brain extraction tool), and head motion correction (MCFLIRT, 6 motion parameters). Images were all linearly realigned to the first T1-weighted image (FLIRT, 6 DOF), registered to the Montreal Neurological Institute 152 (MNI 152) 2-mm standard space (FLIRT, 12 DOF), and corrected for head motion (MCFLIRT, 6 parameters).

Artifacts created by spurious noise and motion can occur in brain imaging, particularly with patients who have difficulty lying still in the scanner for a long period of time. Therefore, in order to limit confounding effect of physiological noises such as cardiac pulsation and modulation associated with the respiration, we used the aCompCor method, which was developed to identify specific patterns of structured noise, and remove them (Behzadi et al., 2007; Kucyi et al., 2013, 2014; Muschelli et al., 2014). The first step of this method segments brain tissue into a component that includes only white matter (WM) and cerebrospinal fluid (CSF), and uses it to model physiological noise (see below). The images were segmented using FSLs segmentation tool (FAST). The WM and CSF components were thresholded to keep only voxels with high probability of being WM and CSF (top 198 cm^3^ and top 20 cm^3^; Chai et al., 2012; Kucyi et al., 2013). Then, a principal component analysis (PCA) was used to model the time series of the noise component with the first five WM and CSF components. This noise component, in addition to the six motion parameters obtained earlier, were then regressed out through its addition in the general linear model as a nuisance component of blood oxygen level dependent (BOLD) time series. Finally, all functional data were spatially smoothed using a 4-mm kernel full-width at half maximum kernel (FWHM) and a 0.005–0.05 Hz band-pass was applied (Rogachov et al., 2016).

### Seed-to-Voxels Whole Brain Analysis

We carefully considered the issue of choosing the PAG seed architecture. As mentioned earlier, the PAG is involved in both pain perception and emotional reaction to a pain and/or stressful stimuli. It has been suggested that the autonomic nervous system is linked to pain modulation deficiency in FM (Chalaye et al., 2012, 2014) and anxiety, hypervigilance and descending pain modulation is likely deficient in FM patients. We chose to seed the whole PAG as has been done in previous studies (Eippert et al., 2009; Kucyi et al., 2013) because: (1) we were interest in the clinical profile of FM; (2) considering the correlation between FC and clinical symptoms, a whole PAG seed was more appropriate than separate left and right PAG seeds (Cifre et al., 2012; Schmidt-Wilcke et al., 2014); (3) this minimized the number of statistical comparisons; and (4) we had no specific hypothesis about the left or right PAG (Kucyi et al., 2014). We are aware of the limitations of this seed considering our previous publication of the columnar organization of the PAG (Coulombe et al., 2016), however, this was best seed to use to answer the aim of the current study.

**Figure 1:**
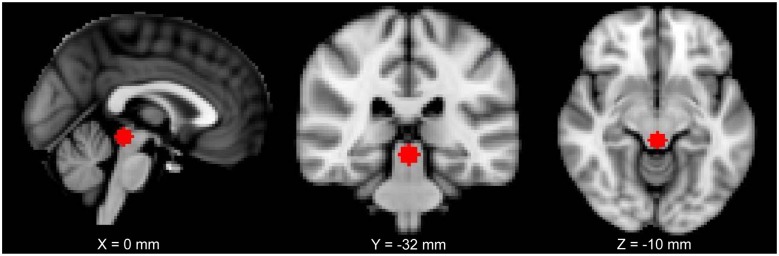
The periaqueductal gray (PAG) seed used in this study shown on a 2 mm Montreal Neurological Institute (MNI) standard space template.

The location of the PAG seed was chosen based on a previous study from our lab (MNI coordinates: 0, −32, −12; size: 6-mm radius sphere; Kucyi et al., 2013; Figure 1). For each subject, the seed was registered to their 2-mm native space (trilinear interpolation to the previously pre-processed image), binarized and the time series of each voxel of the seed was extracted. The BOLD time series was put in relation with the time series of every other voxel of the brain. FMRIB’s FMRI Expert Analysis Tool with FILM (FMRIB’s Improved Linear Model) general linear model using nonlinear registration with a 10-mm wrap was used to analyze each subject. The higher-level analysis used FMRIB’s mixed effects thresholded at *Z* = 2.3 and a cluster-based *P* = 0.05 (Flame 1 + 2).

### Correlation between FC of Identified Clusters with Symptom Severity Metrics

We next further examined the FC of each cluster found to differ between the FM and HC groups (see in the contrast analysis above, and numbered in Table 1). We extracted the values of FC using FSL function “meants”, i.e., the average of the timeseries each set of voxels. These data were correlated, in FM subjects, with the major metrics of negative affect parameters assessed (anxiety, depression, distress, rumination, helplessness, magnification or global catastrophizing score) and the major clinical indices of FM assessed (pain severity, FIQ score normalized). To account for multiple comparisons, we did a Bonferroni correction (α/number of comparison) and, therefore, set our *p*-value at <0.007 for psychological symptoms (7 symptoms to compare) and <0.025 for pain severity/interference (2 scores to compare).

**Table 1:** Peak Montreal Neurological Institute (MNI) coordinates of contrast comparison of periaqueductal gray (PAG) seed functional connectivity (FC) between fibromyalgia (FM) and healthy controls (HC).

		Peak voxel, MNI coordinates		
Region	*Z*-max	*X*	*Y*	*Z*
**FM > HC**			
*Cluster 1*			
L Lingual gyrus (BA17, BA30)	3.8	−24	−62	2
L Posterior cingulate gyrus (BA30)	3.44	−20	−44	−2
L Hippocampus/Cingulate gyrus (BA29, BA30)	3.35	−22	−46	2
L Cuneus/retrosplenial cortex (BA31, BA23)	3.23	−24	−72	4
L Hippocampus	3.06	−20	−38	4
**HC > FM**			
*Cluster 7*			
L Premotor cortex (BA6)	3.42	−28	12	60
L Paracingulate gyrus (BA6, BA32)	3.4	−8	10	46
*Cluster 6*			
L Dorsolateral PFC (BA8)	3.79	−22	26	32
L Paracingulate gyrus (BA32, BA6, BA8)	3.31	−12	36	28
*Cluster 5*			
L Angular gyrus (BA7, BA40)	3.62	−40	−52	42
*Cluster 4*	3.82			
R Angular gyrus (BA7, BA40)	3.82	42	−56	40
R Supramarginal gyrus (BA40)	3.3	58	−38	54
*Cluster 3*			
R Premotor cortex (BA6)	3.65	20	14	58
*Cluster 2*			
R/L Posterior cingulate cortex (BA23)	3.63	4	−24	26
L Precuneous cortex (BA31)	3.51	−4	−44	44
*Cluster 1*			
L Ventrolateral PFC (BA10)	3.33	−22	50	14

*Maximum peak are reported as MNI coordinates (in mm) and corrected for multiple comparisons using family-wise error correction at Z > 2.3 and P < 0.05. BA, Brodmann area; L, left; PFC, prefrontal cortex; R, right*.

## Results

### Description of the FM Population

The demographic profile and symptomology of the FM patients are shown in Table 2. Based on available normative data of the questionnaires used, our patients reported symptoms of moderate severity (Bennett et al., 2009), mild anxiety and depression (normal 0–7, mild 8–10, moderate 11–14 and severe 15–21; Zigmond and Snaith, 1983). All patients had chronic pain and most (20/23) also reported sensory descriptors that are typically associated with neuropathic pain (Bouhassira et al., 2005). The patient PCS scores fell within the 50th percentile, and the subscores of rumination (44th percentile), magnification (63th percentile) and helplessness (55th percentile) place them at moderate risk for development of pain chronicity (Sullivan, 2009). Only four of the 23 FM subjects had PCS scores ≥30 that indicate clinically relevant pain catastrophizing (Sullivan, 2009).

**Table 2:** Clinical manifestation of fibromyalgia in FM group.

Fibromyalgia patients (*n* = 23, possible range)	Mean ± SD	Range [min–max]
Age (years, 19–70)	50.6 ± 8.1	25–62
Brief Pain Inventory (BPI, 0–120)	65.8 ± 17.5	37–93
Subscale	–	–
Pain severity (0–40)	22.3 ± 7.5	7–37
Pain relief (0–10)	3.7 ± 3.6	0–10
Pain interference (0–70)	39.9 ± 12.6	13–61
Pain disability index (PDI, 0–70)	38.7 ± 14.7	0–63
DN4 (score)	5.4 ± 1.6	1–8
Non-neuropathic (# of subject)	3	–
Neuropathic (# of subject)	20	–
Health outcome measured with EQ-5D (5–15)	9.3 ± 1.3	7–11
Hospital Anxiety and Depression Scale	–	–
Subscale	–	–
Anxiety (0–21)	10.4 ± 3.8	2–20
Depression (0–21)	7.3 ± 3.4	2–16
Distress (0–42)	17.7 ± 5.9	4–31
Pain Catastrophization Scale (0–52)	19.8 ± 11.9	2–47
Subscale	–	–
Rumination (0–16)	6.6 ± 4.0	0–15
Helplessness (0–24)	9.0 ± 5.8	0–23
Magnification (0–12)	4.2 ± 2.9	0–11
Fibromyalgia Impact Questionnaire (FIQ) total score (Normalized, 0–100)	60.3 ± 15.8	17.1–92.9
Physical impairment (0–3)	1.4 ± 0.4	0.6–2.2
Feel good (0–7)	4.9 ± 1.9	0–7
Missed work^‡^ (0–7)	0.9 ± 1.5	0–7
Do Work^‡^ (0–10)	6.3 ± 2.3	1–10
Pain (0–10)	7.2 ± 2.2	1–10
Fatigue (0–10)	7.8 ± 2.0	3–10
Rested (0–10)	7.5 ± 2.2	3–10
Stiffness (0–10)	7.8 ± 1.7	4–10
Anxiety (0–10)	4.8 ± 2.9	0–10
Depression (0–10)	3.3 ± 3.1	0–10

*SD indicates standard deviation. ^‡^n = 10 for this criterion*.

### PAG Functional Connectivity in FM and HC

**Figure 2:**
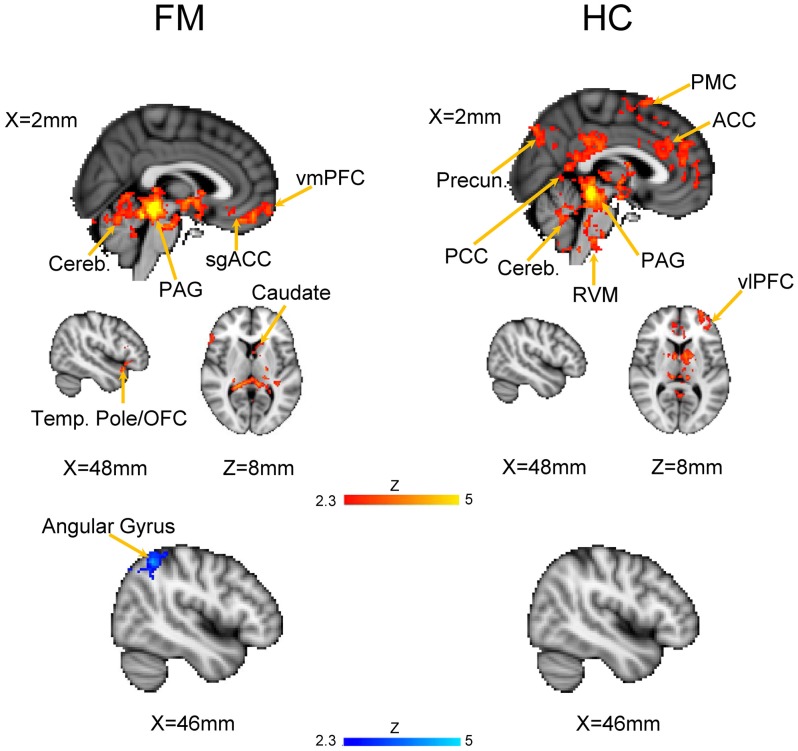
Functional connectivity (FC) of a PAG seed in patients with Fibromyalgia (FM; *n* = 23) and in healthy controls (HC; *n* = 16). Images are thresholded at a whole-brain family wise error rate-corrected *Z* > 2.3; cluster-based *p* < 0.05. Warm colors represent positive correlation, cold colors represent negative correlation. Cereb., cerebellum; vmPFC, ventromedial prefrontal cortex; sgACC, subgenual anterior cinculate cortex; PAG, periaqueducatal gray; OFC, orbitofrontal cortex; Temp. Pole, temporal pole; Precun., precuneus; PCC, posterior cingulate cortex; ACC, anterior cingulate cortex; PMC, premotor cortex; RVM, rostroventral medulla; vlPFC, ventrolateral PFC.

We first evaluated the FC of the PAG in both HC and FM groups (Figure 2, Table 3). Results are expressed as significant clusters of voxels, i.e., few adjacent voxels. In the HC group, we found that the PAG showed significant positive FC with six clusters of brain areas (see Table 3). These clusters included the brainstem, cerebellum, the anterior cingulate cortex (ACC), and the ventrolateral (vlPFC) and ventromedial (vmPFC) parts of the PFC, premotor cortex (PMC), and also to regions in the parietal lobe including the precuneus, angular gyrus (AG) and the posterior cingulate cortex (PCC). There were no significant regions found to have anticorrelated FC to the PAG in the HC group.

**Table 3:** Peak MNI coordinates for brain regions correlated with the PAG seed.

		Peak voxel, MNI coordinates		
Region	*Z*-max	*X*	*Y*	*Z*
**FM**			
*Cluster 3*			
PAG	6.91	4	−34	−12
Cerebellum peduncle	5.65	0	−42	−16
*Cluster 2*			
R/L Ventromedial PFC (BA11)	4.17	4	40	−20
R/L Subgenual ACC/medial PFC (BA11, BA32)	3.33	−8	32	−12
*Cluster 1*			
R Temporal pole (BA38)	3.88	48	18	−16
R Ventrolateral PFC (BA47)	3.78	54	30	4
**HC**			
*Cluster 6*			
PAG	6.8	4	−34	−12
Cerebellum peduncle	4.6	−8	−38	−10
R Posterior cingulate cortex (BA23)	4.55	6	−24	32
R Tail of the caudate nucleus	4.49	26	−30	20
*Cluster 5*			
R/L Premotor cortex (BA6)	4	−30	6	44
L Paracingulate gyrus (BA24, BA32, BA6, BA8)	3.7	−6	22	46
R Ventromedial PFC (BA11, BA10)	3.68	12	48	−10
R ACC (BA32, BA24)	3.58	8	22	30
R Perigenual ACC (BA32)	3.54	12	38	−6
*Cluster 4*			
R Premotor cortex (BA6)	3.78	20	14	58
R Supplementary motor cortex (BA6)	3.53	6	−2	60
*Cluster 3*			
L Ventrolateral PFC (BA10)	3.5	−30	62	6
*Cluster 2*			
L Angular gyrus (BA39, BA40)	3.81	−48	−54	38
L Supramarginal gyrus (BA40)	3.75	−54	−42	20
*Cluster 1*			
R Cerebellum	3.49	32	−62	−38

*Maximum peak are reported as MNI coordinates (in mm) and corrected for multiple comparisons using family-wise error correction at *Z* > 2.3 and *P* < 0.05. BA, Brodmann area; L, left; PFC, prefrontal cortex; R, right*.

In the FM group, there were three clusters with positive FC to the PAG. These clusters encompassed the regions surrounding the PAG such as the mesencephalic reticular formation, subgenual ACC (sgACC), ventromedial and ventrolateral parts of the PFC, and the temporal pole (Figure 2, Table 3). Additionally, the PAG had negative (i.e., anticorrelations) FC with the right AG, supramarginal gyrus and the superior parietal lobule (Figure 2, Table 4).

**Table 4:** Peak MNI coordinates for brain regions anticorrelated with PAG seed.

		Peak voxel, MNI coordinates		
Region	*Z*-max	*X*	*Y*	*Z*
**FM**
*Cluster 1*
R Angular gyrus (BA7, BA 40)	4.16	46	−52	52
R Supramarginal gyrus (BA39, BA 40)	3.61	36	−52	50
R Superior parietal lobule (BA19, BA7)	3.29	26	−78	52
**HC**
No cluster found

*Maximum peak are reported as MNI coordinates (in mm) and corrected for multiple comparisons using family-wise error correction at Z > 2.3 and P < 0.05. BA, Brodmann area; R, right*.

**Figure 3:**
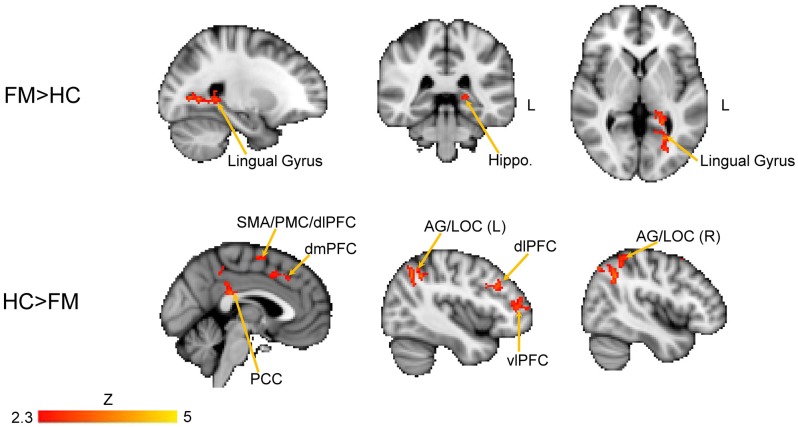
Contrast comparisons between FM and HC. Brain regions with enhanced resting state FC with the PAG in FM (*n* = 23) compared to HC (*n* = 16); FEW-corrected *Z* > 2.3; cluster-based *p* < 0.05. Coordinates for FM > HC, *X* = −24; *Y* = −38, *Z* = 2 (mm). HC > FM, *X* = −2, *Y* = −40, *Z* = 44 (mm). Hippo, hippocampus; PCC, posterior cingulate cortex; PMC, PFC; vlPFC, ventrolateral PFC; dlPFC, dorsolateral PFC; dmPFC, dorsomedial PFC; SMA, supplementary motor area; AG, angular gyrus; LOC, lateral occipital cortex.

We next performed a contrast analysis to determine differences in FC between the HC and FM groups (see Figure 3, Table 1). This analysis revealed a single cluster that had greater PAG FC in the FM group compared to the HC group. This cluster encompassed the left lingual gyrus, PCC, cuneus, retrosplenial cortex and hippocampus. Conversely, compared to the HC group, the FM group had reduced PAG FC with seven clusters of voxels (Table 1) located in the AG/lateral occipital cortex (LOC; bilaterally), PCC, PMC/supplementary motor area (SMA), dorsolateral (dlPFC)- dorsomedial (dmPFC) and vlPFC.

### Correlation between PAG FC and Clinical Manifestation of FM

**Figure 4:**
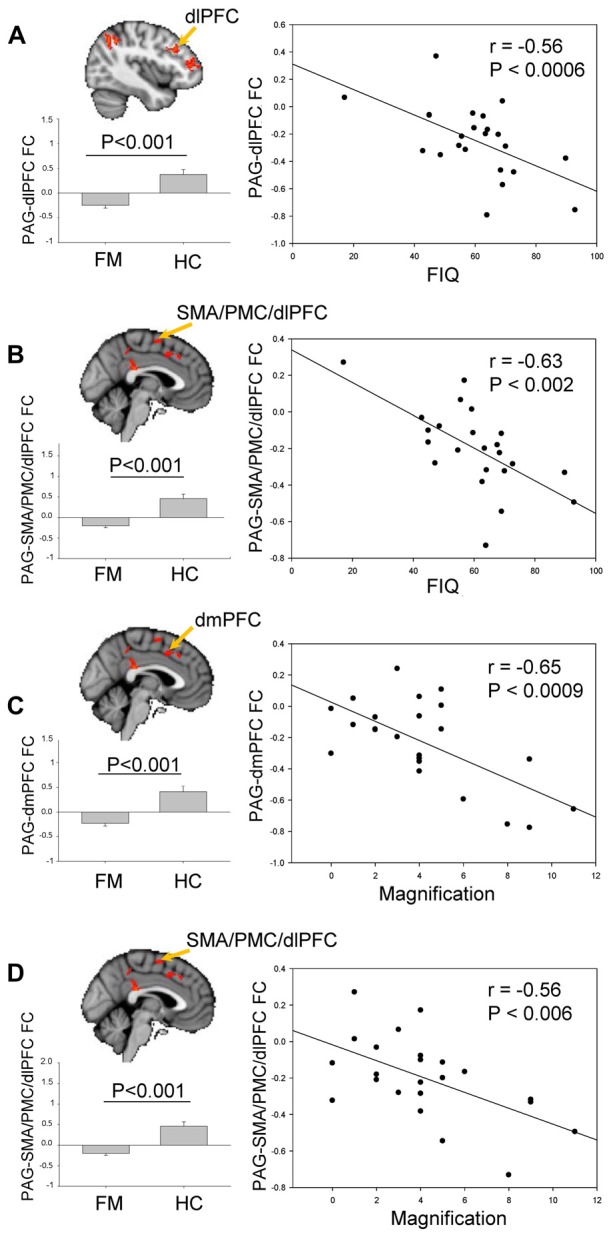
Brain regions exhibiting significant differences of PAG FC between FM and HC (see Figure 3).** (A)** Comparison of PAG-dlPFC FC (cluster 3) between HC and FM, and its correlation with Fibromyalgia Impact questionnaire (FIQ) scores.** (B)** Comparison of PAG-SMA/PMC/dlPFC FC (cluster 6) between HC and FM, and its correlation with FIQ scores. **(C)** Comparison of PAG-dmPFC FC (cluster 7) between HC and FM, and its correlation with the magnification subscale of the Pain Catastrophizing Scale (PCS). **(D)** Comparison of PAG- SMA/PMC/dlPFC FC (cluster 3) between HC and FM, and its correlation with the magnification subscale of the PCS. To control for multiple comparisons, statistical significance was set at *p* < 0.007 because of the seven psychological factors (anxiety, depression, distress, magnification, helplessness, rumination, global PCS score) and at *p* < 0.025 because of the two pain parameters (pain severity, FIQ normalized). PMC, PFC; dlPFC, dorsolateral PFC; dmPFC, dorsomedial PFC; SMA, supplementary motor area.

The contrast comparison between FM and HC (FM > HC and HC > FM) yielded respectively 1 and 7 different clusters (Figure 3, Table 1). These clusters were correlated with pain symptoms and affect in FM subject (Figure 4). The single cluster that showed greater PAG FC in FM compared to HCs (FM > HC, i.e., lingual gyrus, PCC, cuneus, retrosplenial cortex and hippocampus) did not show statistically significant correlations with any of our affective or pain-related parameters. However, for HC > FM, cluster 3 (PMC and SMA) and cluster six dlPFC that showed reduced PAG FC in the FM group both were negatively correlated with the FIQ normalized score (cluster 3: *r* = −0.63, *p* < 0.002; cluster 6: *r* = −0.56, *p* < 0.006)). In addition, cluster 3 (PMC and SMA) and cluster 7 (dmPFC) were both correlated with the magnification subscale of the PCS (cluster 3: *r* = −0.56, *p* < 0.006; cluster 7: *r* = −0.65; *p* < 0.0009). Thus, a greater decrease in FC of these regions was associated with greater magnification of the pain.

## Discussion

In this study, we have demonstrated that FC of the PAG is disrupted in patients with FM compared to HCs, and that weakened connectivity is related to patients’ individual traits and the clinical manifestations of FM. The key findings of our study are compared to HCs, the FM patients had: (1) stronger PAG FC in a single brain cluster that encompasses the lingual gyrus, PCC, cuneus, retrosplenial cortex and hippocampus; (2) weaker PAG FC with regions associated with motor/executive functions, SN and DMN; and (3) decreased PAG FC in the PMC, SMA and PFC, is negatively connected with FM symptoms severity measured with the FIQ and with the magnification sub-score of the PCS. These results are discussed below as potential sites of dysfunction not only in FM, but possibly in other chronic pain diseases.

The various functions of the PAG, and subsequently its dysfunction in chronic conditions, can be understood through its connections to other brain areas and networks. For example, ongoing pain in FM patients has been shown to be negatively correlated with the resting state FC between the PAG and the executive attention network and insula (Pujol et al., 2014). Decreased FC has also been reported between the PAG and the ACC, thalamus and caudate nucleus in FM (Cifre et al., 2012). Furthermore, the (DMN, SN) show dysfunction across many chronic pain diseases, including in FM (Baliki et al., 2008; Napadow et al., 2010; Loggia et al., 2013; Kucyi et al., 2014). These networks may be particularly important in the pathology of FM because of their role in the hypervigilance present in FM (Crombez et al., 2004; Eccleston and Crombez, 2007). Many studies have shown dysfunction along the endocrine axis. For example, low melatonin secretion in FM has been suggested to play a role in the lack of restorative sleep (Wikner et al., 1998), and dysregulation of cortisol (Crofford et al., 1996, 2004; Crofford, 1998) perhaps leading to an allostatic load (sympathetic nervous system dysfunction and a general inability of the system to adapt to changes; Martinez-Lavin and Vargas, 2009; Martinez-Lavin, 2012; Martínez-Martínez et al., 2014). This concept is in line with the proposition that FM might be triggered by stress or altered saliency or interpretation of stimuli (Buskila et al., 2008; Häuser et al., 2011, 2013).

Previous brain imaging studies of FM have focused on pain-related brain abnormalities, evoked brain responses, and on localized regional cerebral blood flow abnormalities (Shokouhi et al., 2016). However, given the wide-range of clinical symptoms in FM, and the likely involvement of brain networks, here we used fMRI to identify variations in whole brain resting state FC of the PAG, and their association with the symptoms prevalent in FM.

It is important to consider the normal connectivity of the PAG to appreciate the dysfunctions found in the FM patients. We found greater FC in the left hippocampal formation, retrosplenial cortex and lingual gyrus in the FM patients. The retrosplenial cortex is adjacent to the caudal part of the corpus callosum (Broadmann area 29–30), and it has connection with the hippocampus formation, the PCC, the precuneus and the lingual gyrus. These regions are involved mostly in spatial memory, orientation and navigation, but some studies associate them to episodic/working memory and emotion-related functions (Sutherland et al., 1988; Maguire, 2001; Warburton et al., 2001; Harker and Whishaw, 2004; Spreng et al., 2009). Interestingly, the retrosplenial cortex has been found to be disrupted across several chronic pain states. For example, Wik et al. (2003) found reduced resting-state cerebral blood flow (rCBF) in the higher retrosplenial cortex of FM patients compared to controls (Wik et al., 2003), and showed that patients had lower rCBF only in the left retrosplenial cortex during acute (“barely tolerable”) pressure pain on a tender-point located above their right elbow for 1 min (Wik et al., 2006). Moreover, another team showed group effects between migraine patients and HC using resting-state FC and a seed located in the anterior insula (Hubbard et al., 2014). They reported increased rs-FC between the anterior insula and a region which appears to be the anterior part of the lingual gyrus, and increased gray matter volume in the left hippocampus in migraineurs. There is also support from animal models for a role of the retrosplenial cortex in chronic pain. For example, decreased metabolism in the retrosplenial cortex was found to predict neuropathic pain in a spinal nerve ligation rat model (Kim et al., 2014). Other animal studies have identified the retrosplenial cortex as being involved in emotional behavior, particularly associated with response to unfamiliar stimuli. Indeed, lesions of the retrosplenial cortex of rats results in increased anxiety and an impaired active avoidance response (Lukoyanov and Lukoyanova, 2006). Thus, in light of these previous studies, our findings suggest that the retrosplenial cortex has a role in a broader range of chronic pain syndromes. Further, given the lack of correlation of this regions with clinical manifestation of FM, we suggest that the role of retrosplenial cortex in chronic pain may be more fundamental to pain itself rather than to a specific negative affect or symptom severity.

Our results showed that the disrupted FCs between the left dlPFC and the right motor associative cortex (SMA/PMC/dlPFC) of FM was negatively correlated with FIQ score, which represent FM symptom severity. dlPFC is a main region of the frontal associative cortex, involved in shifting cognitive set following integration of information of many regions including the limbic system. Also, the dlPFC is particularly known for its central role in working memory holding information, mediating awareness in order to process chronological sequences and self-monitoring. Finally, dlPFC is known to be involved in pain modulation (Lorenz et al., 2003) through its connection with the ACC, the thalamus and the insula, which, when activated, decreased the connectivity between the midbrain and the thalamus resulting in descending pain modulation (Lorenz et al., 2003). Indeed, left dlPFC stimulation using repetitive transcranial magnetic stimulation (rTMS) decreased pain rating (Brighina et al., 2011). This relation between disrupted connectivity between the PAG and dlPFC and increased FIQ score is important because it sheds light on an impaired circuit in FM, which correlates with FM symptomatology. In fact, the involvement of this region does not seem to be specific to FM since a study showed abnormal activation and thinner gray matter of the left DLPFC in chronic low back pain patients compared to HC (Seminowicz et al., 2011). Those results are all in line with our findings, and once again suggest broader implication of this region. The disruption of this region seems to be generalized to multiple forms of chronic pain, and does not seem to be specific to FM or migraines.

Our results showed increased FC between the PAG and many brain regions involved in heteromodal integration of stimuli, which is predominant in the attention and SN, particularly the frontal and parietal association cortex and other regions of the DMN. The connectivity of these regions was not only decreased in FM, it was also correlated with the FIQ score and with the Magnification sub-scale of the PCS. The frontal association cortex plays an important role in sustained and selective attention and allows the brain to engage and disengage when relevant stimuli happen. The PMC and supplementary area are involved in the planning of self-initiated movements and motor learning, but has also been shown to play a role in anticipation of pain, possibly associated with avoidance behavior and catastrophizing (Gracely et al., 2004; Nachev et al., 2008; Kano et al., 2013). In relation to our results, some studies found a role of motor and pre-motor areas in depression. Exner et al. (2009) showed decreased gray matter volume in the right pre-supplementary areas of patients with depressive disorders with melancholic features. This result was associated with psychomotor retardation in these patients. Not only is GMV altered in depression, but so is the WM integrity of pathways connecting SMA, pre-SMA and M1 (Bracht et al., 2012). It has been shown that pain catastrophizing is correlated with development of depression in chronic pain (Keefe et al., 1989). Catastrophization is a maladaptive coping strategy, and it impacts a number of behavioral and neuronal processes involved in the shaping of chronic pain and the disabilities resulting of it.

In FM, disrupted connectivity between the DMN and pre-motor areas could be associated with fear of movement, psychomotor retardation, or maybe the study participants were simply more diligent about the instruction about not to move in the scanner. Furthermore, a recent study showed altered connectivity of the DMN in FM, in relation to the duration of the symptoms and cognitive and emotional processing (Fallon et al., 2016). A relationship between impaired FC of the DMN and the Rumination subscore of the PCS was also observed in temporomandibular syndrome (Kucyi et al., 2014). Interestingly, in our study, rumination was not correlated with any PAG disruption of FC. Disruption of the DMN seems to be present in many chronic pain disorders (Loggia et al., 2013; Kucyi et al., 2014; Hemington et al., 2016), and the involvement of the DMN in the efficiency of descending pain modulation system, as suggested by Kucyi et al. (2014), is very likely.

We note some limitations of our study relate to our choice of the PAG seed. This decision point impacts the results due to the heterogeneity of the PAG as shown in our previous study of the columnar organization of the PAG in HC (Coulombe et al., 2016). However, in our current study, we had a different goal, which was to determine the global involvement of the PAG with symptom severity in FM in general. As such, we did not study certain specifics in detail (e.g., laterality). In the future, it would be interesting to expand upon the current study to specifically detail each PAG column to examine hypotheses about dysfunction of the autonomic nervous system and allostasis in FM. We also note a technical limitation of the current study is the small size of the brainstem and studied regions. Thus, even small seeds and the technical issues of pre-processing are unlikely to be absolutely confined to subdivisions of the PAG and other areas. This issue also motivated our choice of a more global PAG seed.

## Conclusion

Our findings assert the general multi-functionality of the PAG, an important hub that relays information about somatosensory inputs to provide a conscious and emotional perspective, and modulates nociceptive information. Both affective and somatosensory information are important and could influence the pain perception of FM patients. A novel finding was the increased FC in FM between the PAG and the retrosplenial cortex. This region is under-appreciated in chronic pain studies and further studies are needed to understand its specific role in chronic pain conditions. Future studies using a larger sample size could build on our current findings to investigate the columnar organization of the PAG in FM and its involvement in autonomic nervous system which are of interest to understand the pathophysiology of FM.

## Author Contributions

The study was designed by KDD, KStL, DEM and PM-F. DEM and PM-F recruited the patients and did the clinical data assessment. KSL, MS and WRN acquired the imaging data and did some preliminary processing of the data. M-AC created the specific study questions, did the main data analysis and wrote the article with KDD. All authors participated in the editing of the article.

## Conflict of Interest Statement

The authors declare that the research was conducted in the absence of any commercial or financial relationships that could be construed as a potential conflict of interest.

## Footnotes

^1^http://fsl.fmrib.ox.ac.uk/fsl/fslwiki/
